# Risk factors for service use and trends in coverage of different HIV testing and counselling models in northwest Tanzania between 2003 and 2010

**DOI:** 10.1111/tmi.12578

**Published:** 2015-08-25

**Authors:** Caoimhe Cawley, Alison Wringe, Jim Todd, Annabelle Gourlay, Benjamin Clark, Clemens Masesa, Richard Machemba, Georges Reniers, Mark Urassa, Basia Zaba

**Affiliations:** ^1^ Department of Population HealthLondon School of Hygiene and Tropical MedicineLondonUK; ^2^ TAZAMA ProjectNational Institute for Medical ResearchMwanzaTanzania

**Keywords:** HIV testing and counselling, HIV prevention, Tanzania, conseil et dépistage du VIH, prévention du VIH, Tanzanie

## Abstract

**Objectives:**

To investigate the relative effectiveness of different HIV testing and counselling (HTC) services in improving HIV diagnosis rates and increasing HTC coverage in African settings.

**Methods:**

Patient records from three HTC services [community outreach HTC during cohort study rounds (CO‐HTC), walk‐in HTC at the local health centre (WI‐HTC) and antenatal HIV testing (ANC‐HTC)] were linked to records from a community cohort study using a probabilistic record linkage algorithm. Characteristics of linked users of each HTC service were compared to those of cohort participants who did not use the HTC service using logistic regression. Data from three cohort study rounds between 2003 and 2010 were used to assess trends in the proportion of persons testing at different service types.

**Results:**

The adjusted odds ratios for HTC use among men with increasing numbers of sexual partners in the past year, and among HIV‐positive men and women compared to HIV‐negative men and women, were higher at WI‐HTC than at CO‐HTC and ANC‐HTC. Among sero‐survey participants, the largest numbers of HIV‐positive men and women learned their status via CO‐HTC. However, we are likely to have underestimated the numbers diagnosed at WI‐HTC and ANC‐HTC, due to low sensitivity of the probabilistic record linkage algorithm.

**Conclusions:**

Compared to CO‐HTC or ANC‐HTC, WI‐HTC was most likely to attract HIV‐positive men and women, and to attract men with greater numbers of sexual partners. Further research should aim to optimise probabilistic record linkage techniques, and to investigate which types of HTC services most effectively link HIV‐positive people to treatment services relative to the total cost per diagnosis made.

## Abstract (fr)

**Objectifs:**

Etudier l'efficacité relative de différents services de conseil et dépistage du VIH (CDV) dans l'amélioration des taux de diagnostic du VIH et l'augmentation de la couverture du CDV dans les régions africaines.

**Méthodes:**

Les dossiers des patients de trois services CDV (sensibilisation communautaire au CDV durant des cycles d’études de cohorte (CO‐CDV), consultations CDV sans rendez‐vous dans un centre de santé local (WI‐CDV) et dépistage prénatal du VIH (ANC‐CDV)) ont été reliés à des dossiers d'une étude de cohorte communautaire en utilisant un algorithme de couplage probabiliste des dossiers. Les caractéristiques des utilisateurs reliés de chaque service CDV ont été comparées à celles des participants de la cohorte qui n'ont pas utilisé le service CDV, par la régression logistique. Les données de trois cycles d’études de cohorte entre 2003 et 2010 ont été utilisées pour évaluer les tendances de la proportion de personnes testées dans les différents types de service.

**Résultats:**

Les rapports de cotes (odds ratios) ajustés pour l'utilisation du CDV chez les hommes avec un nombre croissant de partenaires sexuels au cours de la dernière année et chez les hommes et les femmes VIH‐positifs par rapport aux hommes et aux femmes séronégatifs, étaient plus élevés dans les services WI‐CDV que dans les services CO‐CDV et ANC‐CDV. Parmi les participants de la surveillance sérologique, la majorité des hommes et des femmes séropositifs ont appris leur statut via les services CO‐CDV. Cependant, il est possible que nous ayons sous‐estimé les nombres diagnostiqués dans les services WI‐CDV et ANC‐HTC, en raison de la faible sensibilité de l'algorithme de couplage probabiliste des dossiers.

**Conclusions:**

Comparés aux services CO‐CDV ou ANC‐CDV, les services WI‐CDV étaient plus susceptibles d'attirer les hommes et les femmes séropositifs, et d'attirer les hommes avec un plus grand nombre de partenaires sexuels. Des recherches supplémentaires devraient viser à optimiser les techniques de couplage probabiliste des dossiers et investiguer quels types de services CDV rallient le plus efficacement les personnes séropositives à des services de traitement en fonction du coût total par diagnostic établi.

## Abstract (es)

**Objetivos:**

Investigar la efectividad relativa de los diferentes servicios de asesoramiento y prueba del VIH (APV) en la mejora de las tasas de diagnóstico del VIH y el aumento de la cobertura de APV en África.

**Métodos:**

Se cruzaron los datos de pacientes de tres servicios de APV (el de extensión comunitaria, realizado durante las visitas programadas a la cohorte de estudio (CO‐APV), el APV a personas que acudieron voluntariamente al centro sanitario local (APV‐V) y el APV prenatal (APV‐PN)), con datos de un estudio comunitario de cohortes utilizando un algoritmo probabilístico de vinculación de registros. Las características de aquellos usuarios vinculados, de cada uno de los servicios de APV, se compararon mediante una regresión logística, con los participantes de la cohorte que no utilizaban los servicios de APV. Se utilizaron los datos de tres rondas de visitas a la cohorte, realizadas entre 2003 y 2010, para evaluar las tendencias en la proporción de personas que se hacían la prueba en diferentes tipos de servicio.

**Resultados:**

La razón de momios ajustada para el uso de APV entre hombres con un número incremental de parejas sexuales en el último año, y entre hombres y mujeres VIH positivos, comparado con hombres y mujeres VIH negativos, era mayor en APV‐V que en CO‐APV y APV‐PN. Entre los participantes de un estudio serológico, el mayor número de hombres y mujeres VIH positivos se enteraban de su estatus via CO‐APV. Sin embargo, es posible que hayamos subestimado el número de diagnósticos en el APV‐V y APV‐PN, debido a la baja sensibilidad del algoritmo probabilístico de vinculación de registros.

**Conclusiones:**

Comparado con CO‐APV o APV‐PN, era más probable que el APV‐V atrajese a los hombres y mujeres VIH positivos, y a los hombres con mayor número de parejas sexuales. En el futuro, la investigación llevada a cabo debería buscar optimizar las técnicas de vinculación probabilística de datos, y averiguar cuales son los tipos de servicios de APV más efectivos para vincular a las personas VIH positivas con los servicios de tratamiento, en relación con el coste total por diagnóstico realizado.

## Introduction

The traditional model of HIV testing and counselling (HTC) service delivery in many countries in sub‐Saharan Africa has been at voluntary counselling and testing (VCT) centres, provided either as stand‐alone services or at clinics attached to health facilities. However, in response to a need to increase HTC uptake in sub‐Saharan Africa 1 and in recognition that alternative models of service delivery may help to reach different population groups, there has been a drive to diversify strategies for HTC service provision 2, 3, 4. These include HTC services offered routinely to pregnant women at antenatal clinics (ANC), or to attendees of outpatient departments such as sexually transmitted infection or tuberculosis clinics (provider‐initiated testing and counselling or PITC) 2, 5. Additional options for service provision include door‐to‐door testing provided to people in their homes, or temporary or mobile outreach HTC units provided to individuals within their communities or places of work 6, 7, 8, 9.

In Tanzania, little is known regarding the relative effectiveness of different HTC models in attracting people with risky behaviours or HIV infection, or in identifying the greatest absolute numbers of HIV‐positive individuals at an early stage of infection – the latter group being particularly important to identify for the treatment as prevention programmes 10. We used community cohort data linked to facility records from three different HTC services in northwest Tanzania [community outreach testing (CO‐HTC), a walk‐in HTC centre at a health facility (WI‐HTC) and an antenatal testing service (ANC‐HTC)], to compare socio‐demographic, behavioural and clinical factors associated with HTC service use. We also assessed trends in the proportion of persons tested at different service types between 2003 and 2010, by HIV status and socio‐demographic characteristics.

## Methods

### Study setting

The Kisesa HIV community cohort study includes seven villages (adult population approximately 15 000 in 2012) and has conducted 28 approximately half‐yearly rounds of demographic surveillance since 1994, collecting information on residence and survival status of household members, pregnancy, births and migration. Seven rounds of serological and behavioural surveillance (sero‐surveys) have been completed every two‐three years over the same period, with eligibility defined as being resident at the last demographic surveillance round and aged 15 or older at the time of the sero‐survey. Participants were invited to a central location in each village to give finger‐prick blood samples for HIV‐research testing without results disclosure, completed an interview questionnaire on health‐ and HIV‐related knowledge and behaviours, and were offered VCT and free medical treatment for health problems 14, 15. Participation in sero‐surveys has declined over time and was 67% (8008/11 946) at the Sero6 round in 2010. HIV prevalence in the study area was estimated at 6.5% in 2010.

### HIV testing and counselling services in the study area

Three HTC services are available in the study area: (i) a community outreach HTC (CO‐HTC) service operates within each village for approximately one month during sero‐surveys, since the fourth round in 2003/4; (ii) a walk‐in HTC (WI‐HTC) clinic has been permanently available at the study area's only health centre (located within the trading centre – approximately 70% of clients are from within the study area) since 2005; and (iii) PITC has been routinely offered to pregnant women attending the health centre ANC since the roll‐out of a prevention to mother‐to‐child transmission (PMTCT) programme in 2008. Antenatal testing (ANC‐HTC) may be carried out in the ANC or WI‐HTC building, dependant on the availability of staff and test‐kit supplies. All HTC services are provided free of charge.

### Data sources – cohort data

During three sero‐surveys in 2003–2004 (Sero4), 2006–2007 (Sero5) and 2010 (Sero6), data on participants’ socio‐demographic characteristics, sexual and health‐seeking behaviours and reported prior use of any HTC services were collected and were used to investigate factors associated with CO‐HTC, WI‐HTC or ANC‐HTC use. HIV status for all study participants was determined using research test results. Area of residence is defined as rural (located away from the main road and between 5 and 10 km from the trading centre containing the health centre offering WI‐HTC and ANC‐HTC), roadside (villages located along the main tarmac road which runs through the study area), or within the trading centre.

### Data sources – HTC data

Data on CO‐HTC use were obtained by deterministically linking unique anonymous identifiers assigned to those using the service to their research study record. Data from the WI‐HTC, including data on ANC‐HTC which occurred within the WI‐HTC building, were double‐entered for the period 2005–2012 (partial data for 2012). A probabilistic record linkage algorithm was developed to match users of the clinic HTC services (WI‐HTC and ANC‐HTC) to cohort study participants, based on measures of similarity (‘match‐scores’) between personal identifiers (name, sex, year of birth, village, sub‐village) in the two data sets 13. The CO‐HTC data set was used as a gold standard to train the record linkage algorithm. All possible cohort matches for each clinic ID were de‐duplicated and trimmed to select the most likely match for each clinic record (see Data S1). The final linked data set available for analyses contained 4046 clinic IDs matched to a cohort participant (linkage rate of 36.8% – 4046/10 994 WI‐HTC and ANC‐HTC clients matched), 1955 of whom (48.3%) were sero‐survey attendees. The final linked data set had low sensitivity (estimated at 17.8% based on the proportion of correctly matched gold‐standard links) but a positive predictive value (PPV) of 68.9%.

### Statistical methods

**Figure 1:**
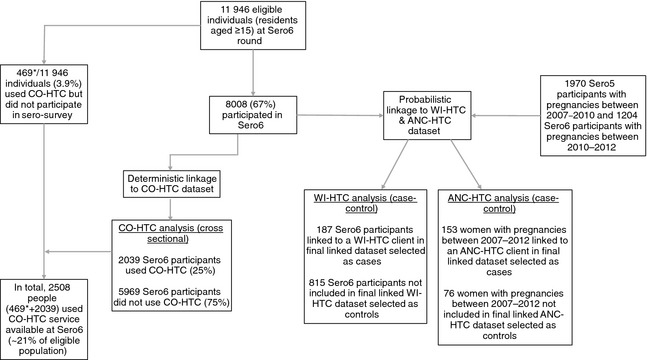
Flow diagram showing participation in Sero6 and individuals included in analyses of CO‐HTC, WI‐HTC and ANC‐HTC use. *This number includes some visitors (non‐residents in the study area) who are not included when calculating the eligible population. Therefore, the total number (and proportion) of eligible (i.e. Kisesa resident) individuals who used CO‐HTC is somewhat smaller than this.

The investigation of risk factors for CO‐HTC was a cross‐sectional analysis comparing the characteristics of all those who used the service compared to all those who did not use the service during Sero6 (Figure 1). For the analysis of WI‐HTC and ANC‐HTC use, a large proportion of cohort participants who were matched to a WI‐HTC or ANC‐HTC client with a low match likelihood were dropped from the data set, because the accuracy of the matches could not be confidently ascertained. The analyses assessing risk factors for WI‐HTC and ANC‐HTC use therefore used a restricted data set (i.e. did not include all sero‐survey participants) and employed case–control methods (Figure 1). For WI‐HTC use, cases were defined as Sero6 participants who were linked to a WI‐HTC client in the final linked data set, with a clinic visit occurring within 2 years of participation in Sero6. For cases with repeat WI‐HTC (18/187), only the first testing visit was used. Controls were selected from among those Sero6 participants who were not included in the final linked data set.

For the analysis of risk factors associated with ANC‐HTC use, women attending either Sero5 or Sero6 were included to increase sample size (Figure 1). Cases were defined as women who participated in Sero5, reported a pregnancy between 2007 and 2010 and were linked to an ANC‐HTC client in the final linked data set with a testing visit within three years of Sero5, or women who participated in Sero6, reported a pregnancy between 2010 and 2012 and were linked to an ANC‐HTC client in the final linked data set with a testing visit within 2 years of Sero6. For cases with repeat ANC‐HTC (3/153), only the first testing visit was used. Controls were selected from Sero5 and Sero6 participants who were not included in the final linked data set and were defined as women who participated in either Sero5 or Sero6, and who reported a pregnancy between 2007 and 2010 (Sero5 attendees) or 2010 and 2012 (Sero6 attendees). A proportion of controls (11/78) (but no cases) participated in both Sero5 and Sero6; these were randomly assigned as a control for one or other round.

Logistic regression models were fitted separately for men and women to identify characteristics independently associated with CO‐HTC, WI‐HTC or ANC‐HTC, using a forward‐fitting approach and including all variables significant in univariable analyses at the *P* ≤ 0.10 level. Likelihood ratio tests were used to assess the inclusion of variables in multivariable models (variable retained if it significantly improved model fit at *P* ≤ 0.10 level). Interactions were explored between HIV status and other characteristics previously found to be strongly associated with HTC use in this setting (area of residence, level of education and previous HTC use) 14, 15. Trends in the proportion of persons testing at different service types were assessed using data on actual or reported HTC use among participants of Sero4, Sero5 and Sero6, by HIV status and socio‐demographic characteristics. All statistical analyses were carried out in Stata 12 (StataCorp, TX, USA).

### Ethical statement

Ethical approval for the activities carried out as part of the Kisesa cohort study, including linkage of WI‐HTC and ANC‐HTC clinic data to the research study data set, was granted by the Tanzanian Medical Research Coordinating Committee and the Ethics Committee of the London School of Hygiene and Tropical Medicine. Participation in sero‐surveys is based on informed consent without disclosure of HIV‐research test results, with a free CO‐HTC service available since Sero4 in 2003–2004 (just prior to the start of the Tanzanian national antiretroviral therapy programme). Verbal consent was obtained during Sero4, due to low literacy rates among the study population. This was witnessed and documented for each participant on their study questionnaire, by a member of the sero‐survey team. During Sero5 and Sero6, written consent was introduced (either a signature or a thumbprint, depending on the participant's writing ability).

## Results

The analysis of CO‐HTC use among Sero6 attendees included 812 men and 1227 women who used HTC, and 2319 men and 3650 women who did not use HTC. For WI‐HTC, there were 75 male and 112 female Sero6 participants who were linked to a WI‐HTC client (cases), and 425 men and 390 women who were controls. For ANC‐HTC, 153 pregnant women were tested and linked to a Sero5 (85) or Sero6 (68) participant, and 76 pregnant women were controls (58 in Sero5 and 31 in Sero6).

### Characteristics of HTC users

In adjusted analyses, men and women aged ≥55 had significantly lower odds of using CO‐HTC and WI‐HTC than those aged 15–24 (Tables 1 and 2). Among women, increasing educational attainment was significantly associated with both CO‐HTC and WI‐HTC but not with ANC‐HTC (Tables 2 and 3). Men and women living in roadside villages or in the trading centre had significantly higher odds of using all types of testing services compared to those living in rural villages, with the exception of WI‐HTC use among women, where the association did not quite reach statistical significance (Tables 1, 2, 3).

**Table 1:** Risk factors for community outreach HTC or walk‐in HTC among men attending Sero6 in 2010^¶,⌂^

	Community outreach HTC (CO‐HTC)^$^						Walk‐in HTC (WI‐HTC)^+^					
	*N*	% using	cOR	95% CI	aOR	95% CI	*N*	% using	cOR	95% CI	aOR	95% CI
Total	3131	25.9					500	15.0				
Age												
15–24	1494	19.7	1		1		167	16.8	1		1	
25–34	458	37.6	2.45	1.95–3.08	1.14	0.84–1.54	43	23.3	1.5	0.67–3.40	0.58	0.21–1.61
35–44	417	35.7	2.27	1.79–2.88	1.14	0.83–1.58	60	35.0	2.67	1.37–5.21	1.3	0.54–3.11
45–54	313	32.6	1.97	1.51–2.58	1.03	0.73–1.47	48	12.5	0.71	0.28–1.83	0.4	0.13–1.22
>55	447	21.3	1.1	0.85–1.43	0.67	0.48–0.94	182	5.5	0.29	0.14–0.61	0.24	0.10–0.59
Area of residence												
Rural	1772	15.8	1		1		278	11.2	1		1	
Roadside	763	36.3	3.04	2.50–3.69	2.61	2.09–3.26	124	20.2	2.01	1.13–3.58	2	1.04–3.85
Trading Centre	596	42.8	3.98	3.24–4.90	3.43	2.72–4.33	98	19.4	1.92	1.03–3.58	1.27	0.62–2.61
Education												
None	453	18.1	1				138	6.5	1			
Primary 1–4	324	25.3	1.53	1.08–2.17			60	6.7	1.02	0.30–3.46		
Primary 5–7	1563	26.7	1.65	1.26–2.14			213	22.1	4.06	1.92–8.59		
Secondary or higher	777	29.1	1.86	1.40–2.47			89	16.9	2.91	1.21–6.97		
Religion												
Catholic	1107	28.2	1				171	18.1	1			
Other Christian	1416	27.0	0.94	0.79–1.13			193	17.6	0.97	0.56–1.65		
Traditional	530	15.8	0.48	0.37–0.63			121	5.8	0.28	0.12–0.65		
Muslim	75	44.0	2	1.25–3.22			15	20.0	1.13	0.30–4.24		
Marital status												
Never married	1466	20.1	0.55	0.46–0.65			176	16.5	1.41	0.82–2.44		
Married monogamous	1325	31.5	1				253	12.3	1			
Married polygamous	128	37.5	1.3	0.89–1.90			26	34.6	3.79	1.56–9.24		
Widowed	46	13.0	0.33	0.14–0.77			23	8.7	0.68	0.15–3.05		
Separated/divorced	97	33.0	1.07	0.69–1.66			18	16.7	1.43	0.39–5.23		
HIV status												
Negative	2947	25.9	1				472	14.2	1			
<3 years since first positive research test	111	29.7	1.21	0.80–1.84			18	27.8	2.32	0.80–6.73		
>3 years since first positive research test	50	24.0	0.91	0.47–1.74			5	40.0	4.03	0.66–24.57		
Reported any previous HCT												
No	2005	19.2	1		1		386	8.8	1		1	
Yes	863	45.5	3.52	2.96–4.19	2.13	1.74–2.61	111	36.9	6.06	3.60–10.22	5.15	2.79–9.50
Has an HIV‐positive relative												
No	2133	24.3	1		1		337	15.4	1			
Yes	479	38.8	1.97	1.60–2.43	1.28	1.00–1.62	60	21.7	1.52	0.77–3.00		
Don't know	274	29.2	1.28	0.97–1.69	1.25	0.92–1.71	103	9.7	0.59	0.29–1.21		
Spouse HIV & VCT use status at Sero6												
No spouse identified	2444	24.9	1		1		354	17.0	1			
Spouse HIV‐neg no VCT	484	19.4	0.73	0.57–0.93	0.56	0.42–0.75	112	6.3	0.33	0.14–0.74		
Spouse HIV‐pos no VCT	29	31.0	1.36	0.62–3.00	0.72	0.30–1.72	7	28.6	1.96	0.37–10.34		
Spouse HIV‐neg used VCT 163	58.3	4.22	3.05–5.84	2.26	1.55–3.28	23	21.7	1.36	0.49–3.81			
Spouse HIV‐pos used VCT 10	60.0	4.53	1.27–16.10	2.82	0.73–10.88	3	33.3	2.45	0.22–27.45			
Age at first sex												
<15	256	21.5	0.54	0.40–0.74	0.64	0.44–0.92	45	15.6	0.82	0.35–1.92		
≥15	1782	33.5	1		1		293	18.4	1			
Never had sex^γ^	825	11.2	0.25	0.20–0.32	0.32	0.23–0.44	–	–	–	–		
Don't know^γ^	237	27.4	0.75	0.55–1.01	0.82	0.58–1.16	158	8.9	0.43	0.23–0.80		
Number of sexual partners in last year												
None	322	21.1	0.58	0.43–0.78	0.65	0.46–0.91	59	10.2	0.85	0.34–2.15	1.17	0.41–3.34
One	1315	31.6	1		1		247	11.7	1		1	
Two or more	640	36.2	1.23	1.01–1.50	1.12	0.89–1.42	91	31.9	3.52	1.96–6.32	2.77	1.41–5.46
Never had sex^γ^	825	11.2	0.27	0.21–0.35	*	*	–	–	–	–	–	–
Don't know^γ^	–	–	–	–	–	–	98	11.2	0.95	0.45–1.99	0.87	0.33–2.28
Had a casual partner in last year												
No	1799	30.4	1				346	14.7	1			
Yes	476	35.3	1.25	1.01–1.55			54	24.1	1.83	0.92–3.66		
Never had sex^γ^	825	11.2	0.29	0.23–0.37			–	–	–	–		
Don't know^γ^	–	–	–	–			98	11.2	0.73	0.37–1.46		
Frequency of condom use with spouse^α^												
Consistent	4	50.0	2.1	0.30–14.98			1	100.0	–	–		
Inconsistent	106	43.4	1.61	1.08–2.41			12	50.0	6.48	1.97–21.30		
Never	1241	32.2	1				247	13.4	1			
No spouse	830	30.2	0.91	0.75–1.10			113	19.5	1.57	0.87–2.84		
Never had sex^γ^	825	11.2	0.26	0.21–0.34			–	–	–	–		
Don't know^γ^	–	–	–	–			98	11.2	0.82	0.40–1.70		
Frequency of condom use with regular partner^β^												
Consistent	39	33.3	1.12	0.57–2.19	0.62	0.30–1.28	6	33.3	2.94	0.52–16.42		
Inconsistent	53	49.1	2.16	1.25–3.73	1.98	1.05–3.73	5	40.0	3.91	0.64–23.97		
Never	116	30.2	0.97	0.64–1.45	1.2	0.75–1.91	15	40.0	3.91	1.34–11.44		
No regular partner	2044	30.9	1		1		371	14.6	1			
Never had sex	825	11.2	0.28	0.22–0.36	*	*	–	–	–	–		
Don't know	–	–	–	–	–	–	98	11.2	0.74	0.37–1.48		

¶, All characteristics as reported at Sero6 in 2010; cOR, crude OR; CI, confidence interval; aOR, adjusted OR; $, cross‐sectional analysis; +, case–control analysis.

⌂, Missing small proportions of data (<5%) for all variables with the exception of area of residence (no missing data), reported any previous HTC (8% missing), has an HIV‐positive relative (8% missing).

γ, Participants reporting ‘never had sex’ at Sero6 reassigned to ‘don't know’ category for WI‐HTC analysis, because these testing visits happened *after* the time of data collection.

*, Omitted because of colinearity; α, First/main spouse among men with more than one spouse; β, First reported regular partner among those with more than one.

**Table 2:** Risk factors for community outreach HTC or walk‐in HTC among women attending Sero6 in 2010^¶,⌂^

	Community outreach HTC (CO‐HTC)^$^						Walk‐in HTC (WI‐HTC)^+^					
	*N*	% using	cOR	95% CI	aOR	95% CI	*N*	% using	cOR	95% CI	aOR	95% CI
Total	4877	25.2					502	22.3				
Age												
15–24	1689	23.3	1		1		78	37.2	1		1	
25–34	1132	32.6	1.59	1.35–1.89	0.98	0.79–1.22	58	58.6	2.39	1.19–4.80	1.76	0.64–4.84
35–44	819	31.3	1.5	1.25–1.81	0.99	0.77–1.25	52	55.8	2.13	1.04–4.35	2.5	0.87–7.21
45–54	516	26.7	1.2	0.96–1.51	0.9	0.67–1.21	59	23.7	0.53	0.25–1.12	0.97	0.32–2.92
>55	720	9.9	0.36	0.28–0.47	0.34	0.23–0.50	255	2.4	0.04	0.02–0.10	0.43	0.11–1.61
Area of residence												
Rural	2493	14.0	1		1		258	20.2	1			
Roadside	1284	34.7	3.27	2.78–3.84	2.96	2.47–3.55	129	27.1	1.48	0.90–2.42		
Trading Centre	1100	39.5	4.02	3.40–4.74	3.81	3.14–4.61	115	21.7	1.1	0.64–1.88		
Education												
None	1829	17.7	1		1		324	10.5	1		1	
Primary 1–4	314	28.3	1.84	1.40–2.41	1.33	0.98–1.80	27	29.6	3.59	1.46–8.83	4.42	1.31–14.95
Primary 5–7	2214	30.1	2	1.72–2.32	1.4	1.17–1.69	121	49.6	8.39	5.07–13.88	2.83	1.33–6.01
Secondary or higher	510	28.6	1.86	1.49–2.34	1.6	1.18–2.16	30	33.3	4.26	1.84–9.86	2.31	0.74–7.27
Religion												
Catholic	2062	26.0	1		1		206	24.3	1			
Other Christian	2433	25.0	0.95	0.83–1.08	1.02	0.88–1.19	207	25.6	1.07	0.69–1.68		
Traditional	253	12.3	0.4	0 27–0 58	0.89	0.57–1.39	67	4.5	0.15	0.04–0.49		
Muslim	117	42.7	2.12	1.45–3.10	1.57	1.02–2.41	20	25.0	1.04	0.36–3.01		
Marital status												
Never married	1008	18.3	0.54	0.45–0.64	0.72	0.50–1.04	49	32.7	0.92	0.47–1.81	0.42	0.07–2.44
Married monogamous	2448	29.4	1		1		180	34.4	1		1	
Married polygamous	402	29.6	1.01	0 80–1 27	1.11	0.86–1.44	44	45.5	1.59	0.81–3.09	1.49	0.66–3.36
Widowed	512	13.1	0.36	0.28–0.47	0.57	0.36–0.89	152	2.0	0.04	0.01–0.13	1.71	0.23–12.50
Separated/divorced	464	28.0	0.94	0.75–1.17	0.97	0.67–1.40	74	14.9	0.33	0.16–0.68	4.35	0.75–25.30
HIV status												
Negative	4502	25.5	1		1		474	20.5	1		1	
<3 years since first	246	24.0	0.92	0.68–1.24	0.59	0.42–0.82	21	57.1	5.18	2.12–12.65	4.14	1.27–13.52
>3 years since first positive research test	113	14.2	0.48	0.28–0.82	0.26	0.15–0.46	6	50.0	3.89	0.77–19.56	11.6	0.68–198.17
Reported any previous HCT												
No	2911	17.5	1		1		396	15.2	1		1	
Yes	1894	37.3	2.82	2.47–3.22	1.49	1.27–1.75	103	48.5	5.28	3.29–8.49	1.89	0 95–3 79
Has an HIV‐positive relative												
No		3478 22.6	1		1		378	19.8	1			
Yes	1148	34.2	1.79	1.54–2.07	1.22	1.03–1.45	92	33.7	2.05	1.24–3.39		
Don't know	199	24.1	1.09	0.78–1.52	0.86	0.59–1.25	32	18.8	0.93	0.37–2.35		
Spouse HIV & VCT use status at Sero6												
No spouse identified	4180	25.3	1		1		425	21.9	1			
Spouse HIV‐neg no VCT	450	14.0	0.48	0.37–0.63	0.55	0.41–0.75	62	24.2	1.14	0.61–2.13		
Spouse HIV‐pos no VCT	37	16.2	0.57	0.24–1.38	0.65	0.26–1.65	5	40.0	2.38	0.39–14.45		
Spouse HIV‐neg used VCT	196	49.5	2.9	2.17–3.87	2.17	1.57–3.00	8	25.0	1.19	0.24–5.99		
Spouse HIV‐pos used VCT	9	44.4	2.37	0.63–8.83	2.49	0.59–10.41	–	–	–	–		
Age at first sex												
<15	467	20.6	0.6	0.48–0.77	0.75	0.57–0.97	78	21.8	0.86	0.47–1.55		
≥15	3431	30.0	1		1		318	24.5	1			
Never had sex^γ^	612	8.7	0.22	0.17–0.30	0.25	0.16–0.39	–	–	–	–		
Don't know^γ^	337	13.9	0.38	0.28–0.52	0.67	0.47–0.95	102	16.7	0.62	0.34–1.10		
Number of sexual partners in last year												
None	858	16.6	0.46	0.38–0.56	1.09	0.75–1.58	225	1.8	0.03	0.01–0.07	0.03	0.00–0.22
One	3261	30.1	1		1		231	40.7	1		1	
Two or more	100	44.0	1.83	1.22–2.73	1.81	1.15–2.86	4	50.0	1.46	0.20–10.53	0.2	0.01–4.04
Never had sex^γ^	612	8.7	0.22	0.16–0.29	*	*	–	–	–	–	–	–
Don't know^γ^	–	–	–	–	–	–	36	33.3	0.73	0.35–1.53	2.51	0.42–14.95
Had a casual partner in last year												
No	4007	27.3	1				457	20.4	1		1	
Yes	211	33.6	1.35	1.01–1.81			9	77.8	13.7	2.80–67.03	10.3	0.72–146.02
Never had sex^γ^	612	8.7	0.25	0.19–0.34			–	–	–	–	–	–
Don't know^γ^	–	–	–	–			36	33.3	1.96	0.94–4.06	*	*
Frequency of condom use with spouse												
Consistent	0	0					–	–	–	–	–	–
Inconsistent	309	39.5	1.65	1.30–2.11			18	77.8	43	12.81–144.19	3.13	0.88–11.07
Never	2458	28.3	1				192	35.4	6.73	3.83–11.84	*	*
No spouse	1389	23.8	0.79	0.68–0.92			239	7.5	1		1	
Never had sex^γ^	612	8.7	0.24	0.18–0.32			–	–	–	–	–	–
Don't know^γ^	–	–	–	–			36	33.3	6.14	2.64–14.27	*	*
Frequency of condom use with regular partner^β^												
Consistent	39	33.3	1.38	0.71–2.69	0.93	0.43–1.99	3	33.3	1.94	0.17–21.62		
Inconsistent	153	45.8	2.33	1.68–3.22	1.64	1.06–2.55	8	62.5	6.46	1.52–27.55		
Never	206	32.0	1.3	0.96–1.76	1.4	0.93–2.12	11	36.4	2.22	0.63–7.74		
No regular partner	3814	26.6	1		1		439	20.5	1			
Never had sex^γ^	612	8.7	0.26	0.20–0.35	*	*	–	–	–	–		
Don't know^γ^	–	–	–	–			36	33.3	1.94	0.93–4.03		

¶, All characteristics as reported at Sero6 in 2010; cOR, crude OR; CI, confidence interval; aOR, adjusted OR; $, cross‐sectional analysis; +, case–control analysis.

⌂, Missing small proportions of data (<3%) for all variables with the exception of area of residence (no missing data).

γ, Participants reporting ‘never had sex’ at Sero6 reassigned to ‘don't know’ category for WI‐HTC analysis, because these testing visits happened *after* the time of data collection.

*, Omitted because of colinearity; β, First reported regular partner among those with more than one.

**Table 3:** Risk factors for ANC‐HTC among women attending Sero5 or Sero6 and reporting pregnancies between 2007 and 2010 (Sero5 attendees) or 2010 and 2012 (Sero6 attendees)^¶,⌂,+^

	*N*	% using	cOR	95% CI	aOR	95% CI
Total	229	66.8				
Age						
15–24	58	60.3	1			
25–34	117	71.8	1.67	0.86–3.24		
35–44	44	68.2	1.41	0.62–3.21		
45–54	10	40.0	0.44	0.11–1.72		
Area of residence						
Rural	114	53.5	1		1	
Roadside	73	86.3	5.47	2.55–11.73	6.25	2.66–14.58
Trading Centre	42	69.1	1.94	0.91–4.11	2.39	0.97–5.86
Education						
None	78	65.4	1			
Primary 1–4	14	78.6	1.94	0.50–7.56		
Primary 5–7	122	68.9	1.17	0.64–2.14		
Secondary or higher	14	42.9	0.4	0.12–1.26		
Religion						
Catholic	82	65.9	1			
Other Christian	122	68.0	1.1	0.61–2.00		
Traditional	17	52.9	0.58	0.20–1.68		
Muslim	8	87.5	3.63	0.43–30.99		
Marital status						
Never married	25	48.0	0.35	0.15–0.83		
Married monogamous	166	72.3	1			
Married polygamous	26	57.7	0.52	0.22–1.22		
Widowed	2	0.0	–	–		
Separated/divorced	10	60.0	0.58	0.16–2.13		
HIV status						
Negative	220	66.8	1			
<3 years since first positive research test	5	60.0	0.74	0.12–4.56		
>3 years since first positive research test	3	66.7	0.99	0.09–11.13		
Reported any previous HCT						
No	162	60.5	1		1	
Yes	66	81.8	2.94	1.46–5.92	2.35	1.07–5.13
Spouse HIV & VCT use status at Sero6						
No spouse identified	167	61.7	1		1	
Spouse HIV‐neg, no VCT	34	82.4	2.9	1.14–7.39	6.58	2.11–20.57
Spouse HIV‐pos, no VCT	2	50.0	0.62	0.04–10.11	0.53	0.02–11.91
Spouse HIV‐neg, used VCT	25	80.0	2.49	0.89–6.95	4.08	1.13–14.80
Spouse HIV‐pos, used VCT	–	–	–	–	–	–
Has an HIV‐positive relative						
No	166	70.5	1		1	
Yes	42	71.4	1.05	0.50–2.21	0.73	0.30–1.78
Don't know	21	28.6	0.17	0.06–0.46	0.18	0.06–0.60
Age at first sex						
<15	11	63.6	0.79	0.22–2.78		
≥15	200	69.0	1			
Don't know	18	44.4	0.36	0.14–0.95		
Number of sexual partners in last year						
None	6	66.7	0.88	0.16–4.93		
One	203	69.5	1			
Two or more	10	50.0	0.44	0.12–1.57		
Don't know	10	30.0	0.19	0.05–0.75		
Had a casual partner in last year						
No	205	67.3	1		1	
Yes	14	85.7	2.91	0.63–13.39	3.37	0.59–19.19
Don't know	10	30.0	0.21	0.05–0.83	0.34	0.07–1.51

¶, Pooled analysis – characteristics as reported at either Sero5 (2006/7) or Sero6 (2010).

⌂, Missing small proportions of data (<1%) for the following variables: education, HIV status, reported any previous HTC, spouse HIV & VCT use status at Sero6.

+, case–control analysis; cOR, crude OR; CI, confidence interval; aOR adjusted OR.

Self‐reported prior HTC was strongly associated with the use of all three testing service types. Men with two or more sexual partners in the last year were significantly more likely to use WI‐HTC compared to men with one partner (aOR: 2.77, 95% CI: 1.41–5.46; Table 1). While a similar trend for was seen for CO‐HTC use, the measure of effect was not as strong nor was the result statistically significant (aOR for men with two or more partners in the last year compared to those with one partner: 1.12, 95% CI: 0.89–1.42; Table 1). Although a larger proportion of women with two or more partners in the last year used WI‐HTC compared to those with one partner (50% *vs*. 41%), this was not statistically significant, likely due to the small number reporting two or more sexual partners (*n* = 4; Table 2). For the analysis of CO‐HTC use, women with two or more sexual partners in the last year were statistically significantly more likely to test compared to those with one partner (aOR: 1.81, 95% CI: 1.15–2.86; Table 2). In adjusted analyses, there was weak evidence (based on a small sample size of nine women, resulting in wide confidence intervals) for an association between having a casual partner in the last year and WI‐HTC use (aOR: 10.25, 95% CI: 0.72–146.02; Table 2), but a similar finding was not observed among CO‐HTC users.

There was some evidence that HIV‐positive individuals were more likely to use WI‐HTC than HIV‐negatives. Among men, the results did not quite reach statistical significance (Table 1); however, women who were HIV‐positive <3 years since first positive research test had higher odds of using WI‐HTC than HIV‐negatives (aOR: 4.14, 95% CI: 1.27–13.52; Table 2). The result was just short of reaching statistical significance for women HIV‐positive ≥3 years since first positive research test, although the trend was in the same direction. In contrast, HIV‐positive men and women were not more likely to use CO‐HTC or ANC‐HTC compared to HIV‐negative individuals (Tables 1, 2, 3). For CO‐HTC use, there was an interaction between HIV status and previous HTC use. Men and women HIV‐positive ≥3 years since first positive research test who reported previous HTC were significantly less likely to use CO‐HTC than HIV‐negative individuals (men: OR: 0.30, 95% CI: 0.12–0.74; women: aOR: 0.18, 95% CI: 0.09–0.36). However, these individuals were not significantly more or less likely to use CO‐HTC if they reported no prior HTC (men: OR: 1.98, 95% CI: 0.75–5.24; women: aOR: 0.68, 95% CI: 0.27–1.72; *P* values for interaction: men: *P* = 0.01, women: *P* = 0.06).

### HIV prevalence by service type

**Figure 2:**
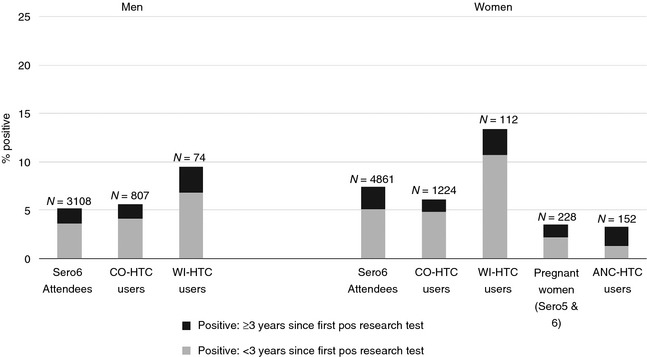
Proportion testing HIV‐positive (based on sero‐survey research test results) by time since first positive research test and HTC service type. *N* represents total sample size, with bars showing percentage of *N* who tested HIV‐positive.

Based on sero‐survey research test results, HIV prevalence was highest among WI‐HTC users (men: 9.5%, women: 13.4%) compared to CO‐HTC users (men: 5.6%, women: 6.1%) or ANC‐HTC users (4.9%) (Figure 2). Among HIV‐positive individuals, differences in the proportions testing at an early stage of infection (<3 years since first positive research test) by testing service type were not statistically significant, but sample sizes were small for WI‐HTC (men: seven positive individuals, women: 15 positive individuals) and ANC‐HTC (five positive women), so results should be interpreted with caution (men: CO‐HTC: 72.1%, WI‐HTC: 71.4%, chi‐square test *P* = 0.9; women: CO‐HTC: 79.5%, WI‐HTC: 80.0%, ANC‐HTC: 40%, chi‐square test *P* = 0.12; Figure 2).

### Trends in the proportion of persons ever tested by HIV status

**Figure 3:**
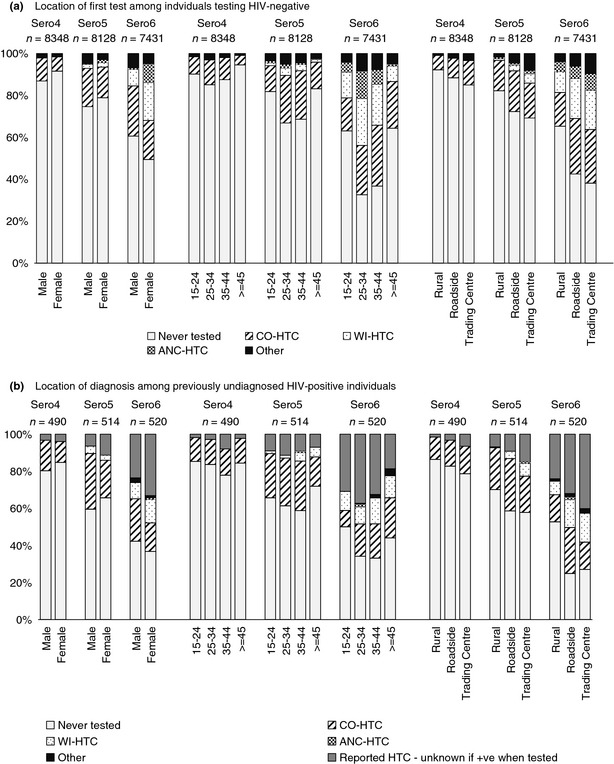
Location of (a) first test for HIV‐negative individuals, (b) diagnosis for previously undiagnosed HIV‐positive individuals (actual or reported HTC use), among attendees of Seros 4, 5 and 6 by sex, age group and area of residence.

The proportion of HIV‐negative individuals receiving their first test at WI‐HTC or ANC‐HTC has grown over time, particularly among women and those aged 25–44 (Figure 3a). By Sero6, similar proportions of HIV‐negative women had first tested at CO‐HTC (18.6%) or WI‐HTC (18.1%), while the greatest proportion of HIV‐negative men had first tested at CO‐HTC (23.9%, *vs*. 7.9% first testing at WI‐HTC). The greatest proportions of HIV‐positive individuals were diagnosed at CO‐HTC. However, we are likely to have underestimated the proportions of HIV‐positive individuals diagnosed at WI‐HTC and ANC‐HTC due to the low sensitivity of the final linked data set, and reliance on reports of previous HTC use for which we did not know the test result. At the later sero‐survey rounds in particular, increasing proportions of HIV‐positive individuals reported previous HTC (at the WI‐HTC, ANC‐HTC or elsewhere – 9.5% of all HIV‐positive individuals at Sero5 and 30.2% of all HIV‐positive individuals at Sero6), but it was unknown whether testing occurred before or after seroconversion (Figure 3b).

## Discussion

Our results revealed that WI‐HTC was more likely to attract men with greater numbers of sexual partners in the last year than CO‐HTC. A similar pattern was not observed among women, although the numbers of women reporting two or more sexual partners in the last year were small, particularly among WI‐HTC users, possibly as a result of social desirability biases 16. This may have affected our ability to detect any association between numbers of sexual partners and WI‐HTC use among women. There was also evidence that WI‐HTC was more likely to attract HIV‐positive men and women than the other testing modalities. This is in agreement with other studies which found that stand‐alone or clinic‐based HTC identified larger proportions of HIV‐positive patients than mobile or outreach testing services 8, 17, 18. Users of client‐initiated WI‐HTC may be motivated by recent exposures or by suspicions that they are HIV‐infected, due to symptoms or death of a partner. Conversely, CO‐HTC and ANC‐HTC represent more passive opportunities to test and therefore may attract relatively fewer high‐risk individuals. However, the aforementioned studies found that outreach testing services were better at facilitating earlier HIV diagnosis clinic‐based services, which tended to diagnose patients at a later stage of infection. We did not find evidence for significant differences in HIV diagnoses by stage of infection by testing service type (Figure 2), but our analyses were limited by small sample sizes, particularly for users of WI‐HTC and ANC‐HTC.

Among sero‐survey participants, the largest numbers of HIV‐positive men and women learned their status via CO‐HTC. However, this may not be true in the wider population, if we had been able to take account of WI‐HTC and/or ANC‐HTC use among individuals who did not attend sero‐surveys. We are also likely to have underestimated the numbers diagnosed at WI‐HTC and ANC‐HTC, due to low linkage sensitivity and reliance on reports of HTC use in the past for which we did not know test results (for 4% of HIV‐positive individuals at Sero4, 10% of HIV‐positive individuals at Sero5 and 30% of HIV‐positive individuals at Sero6). Nevertheless, CO‐HTC is likely to represent an efficient model of service delivery in which large numbers of people were reached in a short period of time. Achieving high HTC coverage is an important objective of HIV prevention programmes in sub‐Saharan Africa, and a number of studies have found that uptake rates are highest when testing is provided as an outreach service 6, 8, 9, 17, 19. One meta‐analysis reported that in 14 studies, most of which were conducted in sub‐Saharan Africa, 87% of individuals accepted mobile outreach HTC when it was offered 9. This is considerably higher than the uptake of CO‐HTC in Kisesa, which was 25% at Sero6. Differences in uptake of outreach HTC between settings may relate to the way in which services are provided, for example whether services are offered independently or as part of a research activity, levels of community mobilisation, and/or differences in the prevalence of HIV‐related stigma between settings. In terms of treatment as prevention, it will be important that HTC services can be regularly accessed, and to understand which services most effectively link HIV‐positive individuals to care and treatment 20.

The three main HTC services in this setting attracted users with different socio‐demographic profiles. Overall, WI‐HTC attracted a greater proportion of women than men, while the proportions of women and men using CO‐HTC were more even (Figure 3). By Sero6 in 2010, a larger proportion of women had ever tested at any HTC service compared to men. These findings concur with other studies which found that men were less likely to use health facility‐based HTC compared to women 18, 21 and that larger numbers of women had ever tested overall 5, 22

In adjusted analyses, WI‐HTC was less likely to attract individuals aged ≥55 compared to those aged 15–24, and less likely to recruit women with no education compared to those with primary education. While similar patterns for age group and level of education were seen for CO‐HTC, the measures of effect were not as strong. A number of studies have shown that outreach testing and some types of PITC reach proportionately older and less educated clients compared to walk‐in HTC 18, 23, 24, and we similarly found that access to CO‐HTC and ANC‐HTC appeared more equitable in terms of these socio‐demographic characteristics compared to WI‐HTC. However, there were inequities in access by area of residence for all testing service types, with the exception of WI‐HTC use among women. It was somewhat surprising that men and women living in rural areas were considerably less likely to use CO‐HTC seeing as this service was provided within the village. Larger distances to the health centre (where HIV care services are available), perceived lack of need, stigma or residual confounding by unmeasured socio‐economic factors may be some of the reasons explaining this finding. Policies that aim to promote and normalise HTC in rural villages may help to increase the uptake of testing in these areas.

Unlike many studies relying on data collected at HTC clinics, a key strength of our analysis was the linkage of community cohort and clinic data, allowing comparison of factors associated with uptake of three different HTC services at the community level. Nevertheless, the final linked data set had low sensitivity (17.8% – many clinic records were dropped during the validation procedures), and this may have introduced some biases which need consideration. Linked individuals included in the WI‐HTC analyses were less likely to be male compared to records not included (33%% *vs*. 37%%, *P* = 0.004). This may have led us to underestimate the proportions of men using WI‐HTC. Linked individuals included in the WI‐HTC and ANC‐HTC analyses were also more likely to be older (*P* < 0.001) and less likely to have secondary education (*P* = 0.002) compared to those not included, which may have led us to underestimate the strength of associations between age and/or educational attainment and WI‐HTC or ANC‐HTC use. WI‐HTC and ANC‐HTC clients included in analyses may also have differed from those not included in other ways which we were unable to measure, and small sample sizes may have prevented us from detecting weaker effects for some risk factors. Nevertheless, our results are broadly in line with previous studies that compared the characteristics of users of similar types of HTC services at other sites in sub‐Saharan Africa 18, 23, 24, giving confidence in our study findings.

We have seen a declining participation in sero‐surveys over time. Declines in participation have been greatest among young men, likely as a result of migration for work. Previous research has shown that migration is associated with a higher risk of HIV infection 25 and also that HIV‐positive individuals are less likely to participate in population‐based research 26, 27, 28. This may have resulted in underestimates of the strength of the associations between HIV status, sexual risk behaviours (numbers of sexual partners, condom use, etc.) and CO‐HTC, WI‐HTC and ANC‐HTC use, particularly among men. However, previous analyses have shown similar factors to be associated with CO‐HTC use over successive rounds of the Kisesa cohort study 14, 15, suggesting that the bias introduced by declining participation is likely to be small. The proportions of individuals who used the CO‐HTC service offered at Sero6 but who did not consent to completing the questionnaire were small (estimated at <4% of all those eligible), and so this is unlikely to have biased the estimates of risk factors associated with CO‐HTC use.

Sensitivity analyses were explored which increased the PPV of the linked WI‐HTC and ANC‐HTC clinic‐cohort data sets from 68.9% to 85.0%. These did not change the overall direction of any of our findings, although in some cases they strengthened associations (as expected given that random error should be reduced in higher PPV data sets), giving confidence in our findings. An additional strength of our study was the ability to explore associations between HIV status and HTC use due to knowledge of HIV status among both testers and non‐testers.

## Conclusions

Of the three services available, the odds of attracting high‐risk men, and HIV‐positive men and women, was greatest at WI‐HTC. Among sero‐survey participants, the largest numbers of HIV‐positive men and women learned their status via CO‐HTC. However, we are likely to have underestimated the numbers diagnosed at WI‐HTC and ANC‐HTC due to low sensitivity of the probabilistic record linkage algorithm. Further research should aim to optimise probabilistic record linkage techniques in order to maximise their sensitivity and positive predictive value and should investigate which types of HTC services most effectively link HIV‐positive people to treatment services relative to the total cost per diagnosis made.

## Supporting information


**Data S1.** Development and validation of the probabilistic record linkage algorithm.
