# Supplementary material for: Risk factors for service use and trends in coverage of different HIV testing and counselling models in northwest Tanzania between 2003 and 2010
Source: Trop Med Int Health. 2015 Aug 25;20(11):1473–87. doi: 10.1111/tmi.12578 (PMC4832370; doi:10.1111/tmi.12578)
Supplement: Supplementary file 1 — Data S1. Development and validation of the probabilistic record linkage algorithm. [file TMI-20-1473-s001.docx]

**Supplementary material – development and validation of the probabilistic record linkage algorithm**

A probabilistic matching algorithm based on personal identifiers (name, sex, year of birth, village, sub-village) was developed to link users of the health centre HTC to cohort study participants. The correspondence between each identifier in the two datasets was given a matching score (e.g., for name, village and sub-village we used the Levenshtein string distance metric, sex was assessed on exact match (true or false), year of birth score was a function of the absolute value of the difference between the two values). A weighted sum of these scores was used to calculate a total score and rank record-pairs according to their likelihood of a match. The algorithm was trained on a gold-standard dataset of participants who used a community-based HTC service and were deterministically linked to cohort study participants using a unique numeric identifier. There were 3,718 record pairs in the gold standard dataset, 338 of which were true matches. The remaining 3,380 records represented intentionally mismatched pairs.

Matching scores were calculated for every combination of record pairs (the Cartesian product of the two datasets). That list was subsequently trimmed by (i) eliminating cohort participants who were recorded as having died before the date of clinic visit (with a buffer of six months around the date of death in order to account for reporting inaccuracies), or cohort participants who left the study area more than a year before the HTC clinic opened in 2005, and (ii) by picking the record pair with the highest match likelihood. Whenever the same cohort match had been selected as the best match for multiple clinic IDs (clinic re-visits by the same client are often given a new ID number because these are mere serial numbers in an HTC logbook), we kept the record-pair with the highest match likelihood.

After de-duplication and elimination of cases based on the residency episode data, the linked dataset was sorted in descending order of match likelihood, and clerical review of every 100^th^ record-pair was undertaken. The review allowed us to identify a score below which matches were doubtful, and records below this threshold were dropped. The final linked dataset available for analyses contained 4,046 clinic IDs matched to a single cohort study participant, representing a linkage rate of 36.8% (4,406/10,994 HTC clients). Of the 4,046 clients linked to a cohort study record, 1,955 (48.3%) were sero-survey attendees.

The final dataset of 1,955 matched records had a relatively low sensitivity: an estimated 17.8% of the 338 true matches in the gold standard dataset were matched probabilistically. At 68.9%, the positive predictive value (PPV) was much higher. At the expense of further loss in sensitivity, the PPV could be increased to 78.5% and 85.0% by imposing more restrictive criteria regarding (i) the overlap between period of residency in the study area and the clinic visit date, and (ii) the match-score on sub-village (sub-village was found to be a good discriminator between true-matches and non-matches in the gold standard data).
